# A “Motivation” model of couple support for digital technology use among rural older adults

**DOI:** 10.3389/fpsyg.2023.1095386

**Published:** 2023-02-03

**Authors:** Jiaojiao Ma, Jie Cui, Qi Zhang

**Affiliations:** School of Media and Communication, Shanghai Jiao Tong University, Shanghai, China

**Keywords:** older adults, technology use, psychological motivations, China, qualitative analysis

## Abstract

Although recent research has demonstrated spouse support for technology use among older adults, by treating them as a homogeneous group, it has overlooked differences caused by socio-demographic dimensions in their digital engagement. Following the approach of the grounded theory and interviewing 15 couples in a developing aging region (Wuzhi County, Henan, China), this study paints a fuller picture of couple support for technology adoption among older adults in terms of their psychological motivations by dividing older adults into two categories: technology supports and technology recipients. The resulting concepts of motivation (e.g., social norms, benefit driven, perceptual elements, and value satisfaction), particularly limiting motivational factors (e.g.,individual status) reveal the psychological mechanisms behind this process and are conceptualized as a “motivation” model of couple support for digital technology use among rural older adults. Our study has strong implications for active and healthy aging, as policymakers can stimulate external motivation for technology adoption among older adults by fostering a sense of family responsibility and social ethos that encourages couples to help each other. In addition, local communities as stakeholders can educate older adults about the usefulness, ease of use, and risk-averse means of digital technology, and satisfy their altruistic and egoistic psychological needs to increase the inner value satisfaction they gain from the couple support process. By doing this, motivation and engagement are thereby increased, and ultimately, technology adoption in disadvantaged socioeconomic groups may be improved.

## 1. Introduction

In most countries, the aging of the population has entered an accelerated phase due to declining fertility rates and longer lifetimes, raising sustained concerns around the world. Therefore, the construction of an active aging society has become a concerned agenda of social and academic discussion for policymakers and researchers. Although the age-based digital divide is deepening, digital technology use has been proved to have potential benefits for addressing social isolation in later life and enhancing well-being among the oldest: connecting older adults with their friends and families (Barbosa Neves et al., 2019), engaging them in group activities (Satariano et al., 2014), and gaining social support by maintaining and extending their social networks (Millard et al., 2018).

Internet accessing among older adults is growing and a great number of studies have been conducted to explore either the impact that digital technology usage on older adults’ lives, as mentioned above, or the ways in which they learn to use it. For the latter, several studies have noted that older adults search for support from a variety of sources, including formal sources (e.g., helplines), younger family (e.g., grandchildren), friends, colleagues, or life spouses to learn and use digital technologies (e.g., using email, online searching, or device set-up; Anderson and Perrin, 2017; Hunsaker et al., 2020). The technology support provided within a couple may be particularly salient and promising given that older adults prefer help provided at home (Friemel, 2016). In addition, helpers can gain a sense of satisfaction providing technology support while recipients may also have greater satisfaction with themselves and knowledge growth when they successfully adopt technologies (Lee et al., 2021). These backgrounds advocate that examining couple support within older adults for digital technology use undoubtedly benefits active and healthy aging in the digital age.

Although the recent research has turned attention to how older adults use mutual support in their own age group as a way to solve technical problems (e.g., Marler and Hargittai, 2022), developing countries, especially the rural areas have not been the focus of such qualitative investigations. In fact, a large body of cross-sectional literature has shown that Internet access is strongly associated with diverse socio-demographic dimensions including gender, age, education, and income, leading to prominent differentiation in the level of individuals, groups, and nations (Friemel, 2016). The rapid increase in the migration of younger people from rural to urban regions also makes the aging and empty-nesters syndrome in rural areas more prominent (Freeman et al., 2020). As the largest developing country, China has 264 million people aged 60 and over, accounting for 18.7% (National Bureau of Statistics of China, 2021). However, the proportion of Chinese older adults (aged 60 and above) using the Internet only increased to 43.2% in 2021, let alone the rural older adults. On the other hand, in highly-developed countries, like the United States, 64% of adults 65 and older already had Internet access in 2016.^1^ Therefore, samples of digital laggers in developing areas are indispensable when painting a fuller picture of technology adoption and use among older adults. Following the vein, this paper aims to investigate:

Q1: What motivates older adults to provide technical support for their spouses in rural China?
Q2: What motivates older adults to seek technical support from their spouses in rural China?

Our study builds a “motivation” model of couple support for digital technology use among older adults, shows that older adults have the ability to act as technology supporters and provide technical support to their spouses, and identifies the motivational factors that older adults consider when seeking technical support, especially some of the limiting factors which may, to some extent, cause the hesitation of older adults to seek support from their spouses. Finally, we propose that the “value satisfaction” motivation and “digital technology use support behavior” in our model are mutually reinforcing.

## 2. Literature review

### 2.1. Older adults and digital technology adoption

Nearly two decades ago, the media often negatively stereotyped older people as technophobic, weak, unknowledgeable, and laggard users due to their age (Kania-Lundholm and Torres, 2017). They are, therefore, more at risk of both social and digital exclusion. However, in recent years things have changed with older adults being more aware of technological benefits and more engaged with digital media (Hunsaker and Hargittai, 2018). The fast development and evolution of mobile devices, the Internet, and social media is changing older adults’ lives in many ways. Older adults are able to contact and share information with families and friends *via* text, digital photo, voice, or video call (Barbosa Neves et al., 2019).

However, perhaps because of not growing up with digital technology, compared with young people, older adults, sometimes referred to as digital immigrants, tend to rely more on the support of others for learning digital skills. Almost three-quarters (73%) of older adults in the United States indicate that they need support when setting up a new device (Anderson and Perrin, 2017). Indeed, compared to strange professional experts, family members or other close ties play key roles in the adoption of digital technologies among older adults because they are considered trusted individuals (Friemel, 2016). For example, Luijkx et al.’s (2015) study indicates that grandchildren play an important role in older adults’ technology adoption because older adults could easily adopt their grandchildren’s enthusiasm and it might eventually lighten the burden on children. On the other hand, those older adults identified as early adopters of technology due to work experiences or lay interest in technology exploration could also be a salient and effective source of digital media support for their peers (Hunsaker et al., 2020). In China, the effective adoption of digital technology by older adults also relies on outside help from families and friends, especially when using a novel device or app (China Internet Network Information Center, 2022). These backgrounds illustrate how older adults as universal recipients and potential supporters work in terms of digital technology adoption.

Previous studies have extensively examined the main psycho-social determinants that influence the adoption of digital technology older adults, which can be categorized into three dimensions: attitudinal (e.g., interest, perceived usefulness, perceived benefit, perceived ease of use, self-efficacy, and confidence with technology), functional (e.g., equipment, access, education, and digital skills), and physical factors (e.g., reduced dexterity, visual difficulties, or cognitive declines; Jokisch et al., 2020). Lee and Coughlin (2015) specifically identified ten factors affecting older adults’ adoption of technology: value, social support, emotion, independence, experience, confidence, usability, affordability, accessibility, and technology support. Barnard et al. (2013) believe that the use of technology will be greatest when the experience extends beyond function and receptivity to emotional responses. In addition, moderate use of digital technologies including social media generates several favorable psycho-social feedback, such as increased levels of happiness and friendship quality (Ward et al., 2018), greater social support (Tifferet, 2020), and reduced levels of depression (Wang et al., 2019). However, given the disadvantaged status of older adults in their digital skills, these studies tend to focus on the motivations of older adults as technology recipients and rarely discuss the motivations of older adults when they act as early adopters and technology providers. This research gap should require special attention today.

China is shifting into an aging society, characterized by its rapid growth, large size, and regional disparity, but more than half of this population has no access to the internet (China Internet Network Information Center, 2022). In response to this issue, the digital inclusion of older adults has become a hot topic in China in recent years (Yang and Du, 2021). On the one hand, the State Council of China (2020) has issued a series of initiatives (e.g., digital devices with bigger texts, brighter colors, and larger buttons) for addressing the difficulties of older adults in using intelligent technology. The government’s commitment to advancement in ICT is also evident from the fact that a strong structure of mobile networks is provided in Chinese rural areas (Chai and Kalyal, 2019). On the other hand, the progress of ICT is providing new ways to meet the demands of older adults, like the recent emergence and increasing adoption of smartphones because older adults are likely to use such easy-to-use devices to buy daily groceries, enjoy short videos, follow the news, and gather information. Indeed, users aged 50 and above account for 27.4 percent of short video users in China (China Population Association, 2021). Since the beginning of the COVID-19 outbreak, the number of Chinese netizens aged 60 years and older has doubled due to the necessity of using smartphones to show health QR codes, scan venue codes, and do nucleic acid tests (Wang and Jia, 2021; China Internet Network Information Center, 2022).

Notably, although these opportunities suggest an overall increase in digital literacy, still accompanied by several major challenges. One challenge is that the digital divide between urban and rural residents is still prevalent in China and other developing countries. Olsson and Viscovi (2016) noted that those who are more educated and financially capable tend to use the Internet more actively for their benefits. In addition, gender is an important factor and older women still lag behind older men in their adoption of digital technology (Kim et al., 2017). Another main challenge is that some older adults confront setbacks when given support by some family member and are embarrassed to seek the same one for technology help again (Peek et al., 2016). Taken together, when promoting the digital inclusion of older adults from developing regions and healthy aging in the post-pandemic era, actions should be taken in response to the psychological factors influencing both digital technology support and receive among older adults to encourage their motivations for engagement. It should be noted that because smartphones are still the most dominant device (99.5%) for older Chinese Internet users while computers are less than 20% (China Internet Network Information Center, 2022), the term digital technology is applied in this study to focus on the smartphone.

### 2.2. Theories of motivation

Theories of motivation could provide an important perspective for studying older adults’ digital technology adoption as they could help us explain human behavior and answer questions such as what are the psychological factors motivating the use of technology?

Self-determination theory, one of the most prominent and tested theories of motivation, explores how two broad categories of motivation foster sustained engagement: intrinsic and extrinsic (Deci and Ryan, 2000). Intrinsic motivation is generated through the pleasure and satisfaction inherent in performing an activity. An increase in intrinsic motivation creates an emotional mood and leads to effective learning (Parayitam et al., 2010). In contrast, extrinsic motivation involves energy unrelated to any sense of enjoyment, which is driven by other means (Deci and Ryan, 1985). Specifically, extrinsic motivation depends on the satisfaction of three basic psychological needs: competence needs (the desire to feel effective in obtaining valuable outcomes), autonomy need (the desire to self-initiate and self-regulate one’s behavior), and relatedness need (the desire to feel connected to others).

In addition, the Technology Acceptance Model (TAM) can successfully predict users’ intentions and behaviors toward new technologies (Davis, 1989). Specifically, a person’s behavioral intention to use technology is influenced by two psychological beliefs: Perceived Usefulness (the extent to which a person believes that using technology will enhance his/her task performance) and Perceived Ease of Use (the extent to which he/she believes that using technology will be effortless). Along these lines, Kwon and Chidambaram (2000) propose a model for cell phone adoption that includes the following factors: demographic and socio-economic factors, ease of use, comprehensiveness, social pressure, perceived usefulness as extrinsic motivation, enjoyment and fun as intrinsic motivation. Specifically, perceived ease of use significantly influences users’ extrinsic and intrinsic motivations, while concerns about cell phone technology negatively affect intrinsic motivations.

It should be noted that human behavioral motivations change and adapt with age. Socioemotional choice theory (Carstensen et al., 1999) explains from a motivational perspective the “paradox of aging” in which subjective well-being is maintained or improved in adulthood (Diener and Suh, 1997) although aging is associated with an objective decline in physical health. This is because, faced with limited time, older adults adaptively reorganize their goal hierarchy so that emotionally meaningful activities in their social networks (e.g., interactions with loved ones) are prioritized over goals, whereas younger adults do not have this preference.

## 3. Methodology

### 3.1. Design

The study applied the constructivist grounded theory, a qualitative methodology most appropriate for research concerned with exploring phenomena given that it allows for the co-construction of theory by scholars and respondents, links the individual with the social context, and advocates a deep analysis of the phenomenon (Mills et al., 2006). Following the approach of the grounded theory, this study first identified our interview guide. Then, we conducted an in-depth, flexible, semi-structured interview and coded the interview texts at three levels. Finally, a theoretical model of the motivation of couple support for digital technology use was constructed.

### 3.2. Data collection

Different studies have adopted different bottom lines to define older adults including people aged 50+ years (Choudrie et al., 2018), 55+ years (Peacock and Künemund, 2007), and 60/65+ years (United Nations, 2019). This study targeted adults who are 50 years old or above, as (1) Chinese rural youth marry and raise children earlier than urban youth, with a high proportion of residents over 50 having three-generation families;^2^ (2) Rural residents over 50 have a low level of education, with only about 10% having a high school education or higher, leading to early retirement age;^3^ and (3) Later life defined by Mental Health Foundation (2018) begins from age 50.

Wuzhi County, Henan Province, China were selected for identifying our participants. Henan is the most populous and agricultural province in central China. Wuzhi County located in the northwest of Henan is in a serious state of aging with 15.8 percent of the older adults and a *per capita* annual disposable income of 27,772 RMB in 2021.^4^ Therefore, the study of the samples in Wuzhi County can represent an aging developing region to some extent.

To ensure maximum heterogeneity of respondents to gather diverse material, we began the interviews by borrowing Brandtzæg et al.’s (2011) four-category user typology, asking respondents to report through self-report which of the following categories their digital skill level falls in: (1) no skill, (2) low skill, (3) mid skill, and (4) expert. We selected respondents covering the four digital skill levels mentioned above by purposive sampling and snowball sampling based on theoretical sampling principles, for a total of 15 couples aged 50–78 years (see Table 1). The studies involving human participants were reviewed and approved by the research team’s university and all participants signed consent forms before taking part. Participants received gifts such as milk, fruit, cooking oil worth about 100 RMB for their participation. Online interviews from May 2022 to June 2022 instead of face-to-face interviews were conducted by voice call influenced by the spread of COVID-19 and lasted on average 45 min.

**Table 1:** Sample characteristics (*N* = 30).

Group	Number	Gender	Age	Occupation	Status	Education	Technology Support
G1	M-1-MWZ	M	50	Poultry sales	Paid work	JH	Two-way
	F-2-SFZ	F	54	Housewife	Unemployed	JH	Two-way
G2	M-3-SXN	M	56	Associate administrator of rural credit cooperatives	Paid work	JH	Two-way
	F-4-MXF	F	55	Housewife	Unemployed	JH	Two-way
G3	M-5-WWS	M	64	Member of the village Committee	Unemployed	PS	One-way
	F-6-WXH	F	62	Worker	Unemployed	None	One-way
G4	M-7-SXW	M	69	Farmer	Unemployed	PS	One-way
	F-8-CGL	F	65	Sanitation worker	Unemployed	None	One-way
G5	M-9-SZC	M	77	Policeman	Unemployed	JH	None
	F-10-SXZ	F	72	Farmer	Unemployed	PS	None
G6	M-11-MXD	M	52	Lamp sales	Paid work	PS	Two-way
	F-12-MXL	F	54	Housewife	Unemployed	None	Two-way
G7	M-13-MZH	M	58	Primary school teacher	Paid work	SH	One-way
	F-14-HYK	F	57	Worker	Paid work	JH	One-way
G8	M-15-MXQ	M	62	High school teacher	Unemployed	SH	One-way
	F-16-HJL	F	63	Housewife	Unemployed	PS	One-way
G9	M-17-WC	M	69	Farmer	Unemployed	PS	One-way
	F-18-SQZ	F	70	Worker	Unemployed	PS	One-way
G10	M-19-MZL	M	73	Construction worker	Unemployed	JH	One-way
	F-20-XM	F	70	Cook	Unemployed	PS	One-way
G11	M-21-WYX	M	78	Farmers	Unemployed	PS	None
	F-21-XJF	F	75	Craftsman	Unemployed	PS	None
G12	M-23-DYK	M	50	Driver	Paid work	SH	One-way
	F-24-MJG	F	52	Ticket seller	Paid work	JH	One-way
G13	M-25-MDB	M	54	Upfitter	Paid work	PS	One-way
	F-26-WHH	F	58	Housewife	Unemployed	PS	One-way
G14	M-27-BXS	M	65	Plumber	Unemployed	JH	None
	F-28-HJ	F	64	Worker	Unemployed	JH	None
G15	M-29-MXL	M	72	Electric vehicle sales	Unemployed	PS	None
	F-30-SQF	F	70	Fruit sales	Unemployed	PS	None

F: female; M: male; PS: primary school; JH: junior high school; SH: senior high school.

Although semi-structured interview guides were provided, participants were encouraged to ask additional questions. The interview guide contained probing questions addressing the following themes: (a) personal information of the interviewees, (b) interviewees’ use of digital devices, (c) the motivations for (not) receiving/providing digital technology support from the partner, and (e) the feelings after receiving/providing digital technology support from the partner. All questions were open-ended allowing interviewees to discuss their own experiences and their perceptions of the couple’s support for digital technology use. According to the grounded approach, through the analysis of the initial interviews, we found that some older couples would help each other to learn digital technology, so we adjusted our sampling to add some couples who help each other in order to explore more patterns in couple support for digital technology use and achieve a saturation of information on the topic. During this process, interview transcripts were continuously examined by the researchers. Interviewing stopped when data reached saturation, namely, no more unique themes or properties of the pattern emerged and data became repetitive.

The education levels of the respondents were diverse, such as primary, middle and high school. Nearly half (46.7 percent) of respondents had a primary school degree or below. Only 10 percent have a high school degree. The occupations of the respondents were also diverse, such as housewife, worker, farmer, teacher, driver, etc. Among 30 participants, 73% owned smartphones and 20% owned a desktop or laptop computers. Three participants (10%) who used digital media frequently due to their jobs indicated that they were fairly skilled. In terms of mobile application usage, 80% of participants used at least one application. The two most frequently used applications were WeChat and Douyin (Chinses version of TikTok). Phone calls, chatting *via* Wechat, and watching short videos on Douyin were the three most frequently used activities. As for the support behavior, to more fully explore the psychological motivations behind older couples’ technical support behavior, we divided 15 couples’ technical support behavior into the following three patterns: (1) one-way support: eight couples provided technical support to their spouses in only one direction, but their spouses did not provide technical support to them in turn; (2) two-way support: three couples provided two-way couples’ support, i.e., both couples provided technical support to each other; and (3) none: no technical support occurred between four couples.

### 3.3. Data analysis

According to the grounded theory, researchers gathered information, coded and sorted data, identified themes and relationships, and finally attempted to propose a conceptual theory (Starks and Trinidad, 2007). In this study, we performed open coding, axial coding, and selective coding with the interview text as the material with the help of NVivo12.

*Open coding*. Open coding is the first level of coding in grounded theory, in which we repeatedly examined line-by-line transcriptions of the interviews. During this phase, irrelevant content and original concepts with low repetition were eliminated, and the 15 most commonly appearing or most significant initial codes were created (see Table 2).

**Table 2:** Open coding.

Number	Initial scope	Raw data representative statements
1	Organizational norms	Most of the families around me are two couples helping each other, and if I do not teach her, it looks like I’m irresponsible.
2	Moral code	We are a family and it is my responsibility to teach her.
		I know more than she does, so I’m obligated to teach her.
3	Evaluation of spouse’s ability	She dislikes the small font on her phone and the mobile applications have too many features for her to adapt.
4	Self-experience evaluation	I have been using smartphones for 10 years, so I have a lot of experience.
5	Altruistic motivation	She is eager to learn about her children’s life outside, and after learning to use her smartphone, she can find out what her children are up to from the photos and texts in her circle of friends.
6	Egoistic motivation	Previously, I was responsible for paying online living expenses and other expenses in the family. After teaching my wife this mobile payment function, she can take care of this job. This will reduce my work in the family.
7	Physical dysfunction	I have poor eyesight and cannot read the words on my phone.
8	Class identity	I am not well educated and cannot keep up with the pace of development of this era.
		We are a poor couple who cannot afford to use smartphones.
9	Intimacy evaluation	He rarely communicates with me when he has a question, so we both generally do not ask each other questions.
10	Proxy support	My son knows I want to enjoy watching country-themed TV shows and will download them for me directly on my phone. So, I can use my smartphone without learning how to use it.
11	Perceived usefulness	I cook and take care of my grandchildren all day, so I do not need to use smartphone at all.
12	Perceived ease of use	Nowadays, the various software updates on the phone are so fast that it is difficult for me to learn.
13	Perceived riskiness	There are so many examples of cell phone fraud. I’m worried about my money being cheated.
14	Internal value	I used to only use my phone to make and receive phone calls, but now I’m using WeChat to video chat with my family and friends after I learned how to do it. I feel very confident and confident in front of my daughter and my friends.
15	Social value	I can now even teach my peers when I master the skills.

*Axial coding*. We used axial coding, which is a more systematic coding process relating code categories and properties to each other through inductive and deductive thinking. Spontaneous memo writing was conducted as we analyzed the data, themes, and connections looking for patterns. Through a comparative analysis of the initial 15 codes, seven main categories were created (see Table 3).

**Table 3:** Axial coding.

Main category	Sub-category	Supplementary explanation
Social norms	Organizational norms	Whether the grassroots mass self-governance organization where the TS is located has village rules and norms for technology support between couples, these organizational norms make TS proactive in providing technology assistance.
	Moral code	In the social environment, TS naturally developed the moral perception that they should provide technology support to their spouses.
Capability assessment	Evaluation of spouse’s ability	The TS evaluates the spouse’s ability to adapt, psychological quality, and other aspects of competence before providing technology assistance.
	Self-experience evaluation	TS’s objective evaluation of self-experience and skill level.
Benefit driven	Altruistic motivation	Whether the act of technology assistance is beneficial to TR.
	Egoistic motivation	Whether the act of technology assistance is beneficial to TS.
Individual status	Physical dysfunction	TR’s evaluations of their age, memory level, visual acuity, and other physical functions.
	Class identity	TR’s objective evaluation of his or her education level, professional status, and economic income.
Relationship support	Intimacy evaluation	The quality and stability of the intimate relationship between TS and TR.
	Proxy support	Whether TR has access to social support from spouses, children, and people around him/her.
Perceptual elements	Perceived usefulness	The extent to which TR believes that using smartphones can facilitate personal life and increase life satisfaction
	Perceived Ease of Use	TR perceived the level of difficulty in learning to use a smartphone. If it perceives that learning a smartphone is easier, then the more likely it is to seek both help and support.
	Perceived riskiness	TS perceives riskiness in the use of smartphones, which can lead to crises such as leakage of personal privacy or loss of property.
Value satisfaction	Internal value	Both the TS and the TR experience internal pleasure, pride, and self-satisfaction in helping each other, and these value experiences, in turn, contribute to the occurrence of digital technology support behaviors.
	Social value	After the technology support behavior occurs, both the TS and the TR gain external recognition and a sense of accomplishment, In turn, this value experience contributes to the re-occurrence of digital technology support behaviors.

TS = technology supporter; TR = technology recipient.

*Selective coding*. The third stage was selective coding where we sorted out the connections and hierarchies among the seven main categories and integrated them into a “Motivation” model of couple support for digital technology use (see Section 4.4). Finally, we performed a theoretical saturation test, and the results showed that no new explanatory factors emerged, indicating that our model reached the theoretical saturation.

## 4. Results

In this section, we present findings related to the research question. We will first describe the motivations of technology supporters to provide technical support to their spouses, second discuss the motivations of technology recipients to seek help from their spouses, third discuss the findings on value satisfaction motivation, and finally constructed a “Motivation” model of couple support for digital technology use that may guide future research efforts in this area.

### 4.1. Motivations to provide technology support: Social norms, capability assessment, and benefit driven

The first research question focuses on identifying the motivations of older adults, as supporters of digital technology, to provide support to their spouses. Three main categories of motivations are identified: social norms, capability assessment, and benefit driven.

*Social norms* consist primarily of organizational norms and moral codes. First, many interviewees who provided technology support indicated that whether they provided technology support for their spouses would be influenced by organizational norms. Organizational norms focus on village rules and management statutes established by grassroots mass self-governance organizations.^5^ For example, an older adult who had never provided digital technology support for her spouse had this to say:

*“No one is asking…The rural grassroots where I live does not require me to teach my spouse, so I don’t think it’s my responsibility.”* (M-9-SZC)

Second, although there are older adults who believe that the matter of teaching a spouse to use a smartphone should occur in a context where it is prescribed by social organizations or strongly promoted by society, there are exceptions where some older adults indicate that they provide technology use support to their spouses under the influence of moral code. In this context, morality refers to the cognitive perception and inner experience of older adults, and this conceptual form guides their behavior. This moral code arises naturally in the social environment and is not developed by any subject or organization. For example, one older adult said:

*“It’s my responsibility to help my spouse learn to use the smartphone thing because we are a family.”* (F-8-CGL)

The above two stories illustrate that older adults in the role of technology supporters are primarily influenced by social norms when considering whether they should provide digital technology support to their spouses. Further, we found that it is reassuring to note that some Chinese older adults, influenced by their personal ethical perceptions, believe that their personal obligations include “taking the initiative to help their wives or husbands at home, especially in learning and using smartphones.” The influence of such attitudes may promote digital technology use support behaviors among older couples.

*Capability assessment* is a factor that technology supporters consider when providing digital technology use support, which consists of two aspects: evaluation of the spouse’s ability and self-experience evaluation. First, for technology supporters, older adults evaluate their spouse’s ability when considering whether to provide support for their spouses. The evaluation of a spouse’s ability focuses on three dimensions: motivation to learn, adaptability, and psychological quality. When older adults find that their spouse is less motivated to learn about smartphones, this reduces the willingness of older adults to provide support to their spouse. One older adult gave this example:

*“In using smartphones it is often necessary to submit various types of verification codes, etc. My spouse thinks this operation is complicated. She has no desire to learn and I will not teach her.”* (M-21-WYX)

Second, older adults’ accumulated self-experience also plays an important role. Before providing technology support to their spouses, older adults assess their own technology skill level. This increases the likelihood of providing support to a spouse if the older adult has provided similar technology support to others. One older adult cited this example:

*“My peers around me often learn from me about cell phone use, so I have more experience. I am happy to share these experiences with my spouse.”* (M-5-WWS)

Further exploration suggests that the relative technology level ratings between older adults and their spouses also affect the achievability of digital technology support. Older adults with lower technology levels than their spouses, often do not feel equipped to provide support to their spouses.

*“I’m not as good at using my smartphone as my spouse, so how am I going to teach her?”* (F-16-HJL)

This difference in relative technology level indirectly leads older adults to assign a negative meaning to personal technology level, i.e., that the individual is not good enough or qualified enough to teach their spouse.

*Benefit driven* refers to the actual behavior of technology supporters pursuing the realization of personal or spousal benefits that generate technology use support. The benefit driven mainly consist of two parts: altruistic motivation and egoistic motivation.

First, altruistic motivation refers to the idea that older adults, as technology supporters, enhance the value and benefits of their spouse’s digital capital by helping them acquire digital skills to meet their spouse’s needs for entertainment and leisure, shopping and consumption using smartphones. During the interview process, altruistic motivation was repeatedly mentioned by older adults:

*“Nowadays, rural areas have to show QR codes to do nucleic acid testing, which is inconvenient if she doesn't know how. By me teaching her to learn smartphones, she now learns to read the notices sent in the WeChat group and can work well with the work related to epidemic prevention and control in our villages.”* (M-3-SXN)

Second, the egoistic motivation is mainly manifested in the fact that in order to satisfy their ego needs, older adults will help and support their spouses in smartphone use. For example, in the interview, an older adult respondent stated:

*“My daughter is in college and usually gives me WeChat video calls often. When I was working, I couldn’t reply to my daughter's messages as soon as possible. Therefore, I want to teach my wife to use a smartphone so that she can care for my daughter well and I can be more at ease with my work.”* (M-1-MWZ)

### 4.2. Motivations to seek technology support: Individual status, relationship support, and perceptual elements

As for the second research question, we identify the following main motivations for older adults who seek technical support from their spouses: individual status, relationship support and perceptual elements.

*Individual status* is mainly composed of physical dysfunction and class identity. First, as older adults age, they experience physical dysfunction such as declining vision and poor memory levels, which makes it more difficult for them to master digital technology and limits their willingness to learn about smartphones. One older adult mentioned:

*“I’m older and suffer from high blood pressure, heart disease, etc. Looking at my phone for a long time, my eyes not only hurt, but my blood pressure also rises.”* (F-21-XJF)

Similar cognitive evaluations reduce the motivation and enthusiasm of older adults to use the Internet. In the aging process, the main issues of physical decline in older adults include decreased visual sensitivity, hearing loss, and memory loss. Currently, under the influence of weakened physical functions, older adults may perceive that they have great obstacles in using smartphones, which will reduce their motivation to seek technology help.

Second, older adult’s perception of their personal class identity also affects their willingness to seek technology help. This class identity stems from two sub-dimensions: educational attainment and personal economic level. Some older adults highlighted personal deficits in education level and income status. For example:

*“I merely have an elementary school education, low literacy level, and little knowledge of the Internet and information technology.”* (M-29-MXL)

*“My family’s financial situation is not that good, smartphones are so expensive that I can't afford to buy them.”* (F-30-SQF)

It can be seen that the literacy level limits the old adults’ enthusiasm for Internet use. Because the market price of smartphones is higher than that of senior phones, and internet usage requires a certain fee, older adults are less willing to use smartphones under the constraints of their personal economic level, which also limits the possibility of seeking technology support from their spouses.

*Relational support* consists mainly of intimacy evaluation and proxy support. First, the study found that older adults assess the intimacy between them and their spouses. In particular, digital technology use support occurs more often when couples have a strong marital relationship and have less conflict in their lives. One older adult mentioned:

*“Without my wife’s support and help, I couldn’t have learned to post short videos on the DouYin platform so quickly. We have a good relationship between the two of us. Sometimes she helps me videotape some scenes of my work, and I then edit and add music and post them on the Internet.”* (M-1-MWZ)

Another older adult agreed that the quality of intimate evaluation influences the occurrence of supporting behaviors. In addition, she noted that it is important for couples to have strong conflict management skills in their intimate relationships, which would provide an effective guarantee that digital technology support conversations will occur:

*“Once, I asked my spouse for advice about how to make a video call via WeChat. After he taught me for five minutes, I still hadn't learned, so he got a little impatient. This led to an argument between us.”* (F-28-HJ)

This interviewer also indicated that frequent arguments between couples in digital technology support can affect the likelihood of technology support help occurring. This enlightens us to focus on how to optimize the relationship between older couples in order to facilitate the occurrence of digital technology support behaviors.

Second, the study also found that when there are children within the family who provide adequate social support for older adults, this can result in older adults believing that they can rely solely on their children and thus being reluctant to seek support from their spouses. This study calls this phenomenon proxy support, where the older person’s child or the more digitally skilled member of the family participates in digital activities instead of the older person. For example:

*“I live with my daughter and when I go to the hospital, I ask her to help me complete my online clinic appointments and registration. I don’t need to learn how to use the cell phone.”* (F-10-SXZ)

When children within the family raise enough social support for the older person, this can, to some extent, make the older person dependent and thus less willing to learn digital technology and thus integrate into the digital society. Similarly, the over-dependence of older adults on their spouses can also lead to a decrease in the occurrence of helping behaviors. One older adult used the nickname “old boy” to describe her spouse when presenting and praising her husband for his help.

*“My husband works in a job that always requires the use of computers, cell phones, and other smart devices, which makes him very knowledgeable about the various functions of cell phones. Moreover, he often posts work summaries and personal calligraphy works on a content community platform called ‘Mei Pian’ (a online social community for older adults) and forwards the links to his circle of friends. So when I want to post photos of my daily life on the Internet as he does, he can do it for me directly.”* (F-6-WXH)

*The perceptual elements* consist of perceived usefulness, perceived ease of use, and perceived risk. First, perceived usefulness emphasizes the extent to which older adults find using smartphones personally useful. Although smartphones, as new technological tools, have far more functions than the old cell phones that could only make phone calls and send text messages, older adults’ perceptions of the usefulness of cell phone functions to them personally are different. This perception and evaluation can influence whether older adults seek support from their spouses. For example, older adults do not seek support for technology use from their spouses when they believe that using a smartphone is not personally enhancing or helpful. One respondent stated:

*“I don’t need any of the features other than the need to make phone calls to my family through my phone.”* (M-21-WYX)

Second, perceived ease of use refers to the extent to which a person perceives using a smartphone as effortless and not difficult. Older people’s evaluation of the degree of difficulty in using their smartphones can also affect their motivation to seek support. Currently, the diversified functional design of shopping, internet taxi, and ordering food on smartphones brings convenience and speed to the younger group, but the complexity of operational settings makes told adults less willing to use them. One old adult expressed that she thinks some functions on her cell phone is complicated to operate, which will lead her to be reluctant to seek support from her spouse. At the same time, she expressed her expectation for the aging-friendly modification and design of cell phone functions to us:

*“Nowadays, technology is developing so fast and the functions of cell phones are changing day by day. If there are some cell phone brands, that can design specially for old adults use of cell phones, especially some software and features will be age-appropriate optimization. That would be great.”* (F-6-WXH)

Third, perceived riskiness is the risk that a person perceives in the process of using a smartphone that the information or their own behavior poses. The risks perceived by older adults in the process of using smartphones include phone malfunction, information leakage, or internet fraud. This individual risk perception can have an impact on the behavioral intention of older adults to use smartphones. One participant said:

*“Nowadays, the news says that our old adult group is the main victim of telecom fraud. Moreover, smartphones have some hidden battery problems, charging heat and flammability, etc. All these risks and hazards deepen our concern about using smart devices.”* (F-24-MJG)

It is evident that the occurrence of digital fraud cases has created a sense of mistrust in the use of smartphones among older adults, thus, reducing the willingness to use smartphones and to seek support for the use of companion technology. This risk perception evaluation also makes it difficult for digital learning and application among older adults to take place or to achieve the expected results.

### 4.3. Two-Way drivers: Value satisfaction

*Value satisfaction* emphasizes a positive emotional experience resulting from the joint creation of value by both stakeholders (the technology supporter and the technology recipient), and this satisfaction is the driving force that supports the continued participation of both parties in digital technology use support. Based on interviews with older adults, value satisfaction is summarized into two aspects: internal value and social value.

First, internal value mainly emphasizes the inner feelings of older adults after receiving digital technology support, and in essence, older adults develop a sense of self-satisfaction and self-respect. For example, two older adults, after receiving technology support from their spouses, stated that:

*“I have now learned to use my cell phone to pay my monthly electricity bill. This has made my daily life more convenient and I am very satisfied.”* (M-17-WC)

*“After I learned to use my cell phone to send red packets, I don't have to envy other people either. I feel very proud.”* (F-26-WHH)

Second, social value is the positive social evaluation that older adults experience in the process of technology help. For both the technology supporter and the technology recipient, through the use of the smartphone, both parties reaped external recognition and good evaluations and gained meaning in their lives from society. For example:

*When teaching my spouse, a sense of accomplishment came over me, as if I had become a teacher. After teaching her, when she went to the supermarket to pay again, she was comfortable using Alipay to pay. Some of the friends of her age are envious of her.”* (M-19-MZL)

*“I heard from my spouse that we can earn money by downloading video software to watch short videos posted on the platform. After I let my spouse teach me to learn, now, people around me are also learning how this technique from me and I feel a sense of accomplishment.”* (M-7-SXW)

Our interview further suggests that for technology supporters, motivated by value satisfaction, older adults turn to teach spouses to learn to use a smartphone into a purposeful and proactive behavior, and in this context, older adults will help their spouse again. For older adults who are technology recipients, after learning to use a smartphone, they form deep memories to generate good experiences, and this value satisfaction inspires a willingness to learn further and a willingness to engage in digital technology-enhancing behaviors and activities with greater enthusiasm. For example:

*“This interactive behavior about digital technology use support has enhanced our relationship as a couple and improved our happiness in life. As a result, I have recently been teaching my spouse how to download videos and be able to watch this technology offline.”* (M-13-MZH)

*“Next, I want to learn from my spouse about how to prevent online fraud. For example, for the nuisance calls I often receive on my phone, I would like to learn how to set the features so that I can use my smartphone more safely.”* (F-14-HYK)

It can be seen that value satisfaction is both the end point of digital technology use support behavior between an old couple and the starting point for the next occurrence of digital technology support behavior at the same time. After the digital support behavior occurs, the technology supporter and technology recipient will have the emotional experience of value satisfaction, and this value satisfaction will have a strong driving effect on the behavior of both spouses, thus promoting the re-occurrence of the technology support behavior. In other words, A two-way cycle is formed between the emotional experience of “value satisfaction” and the “digital technology use support” behavior.

### 4.4. Building theoretical model

**Figure 1:**
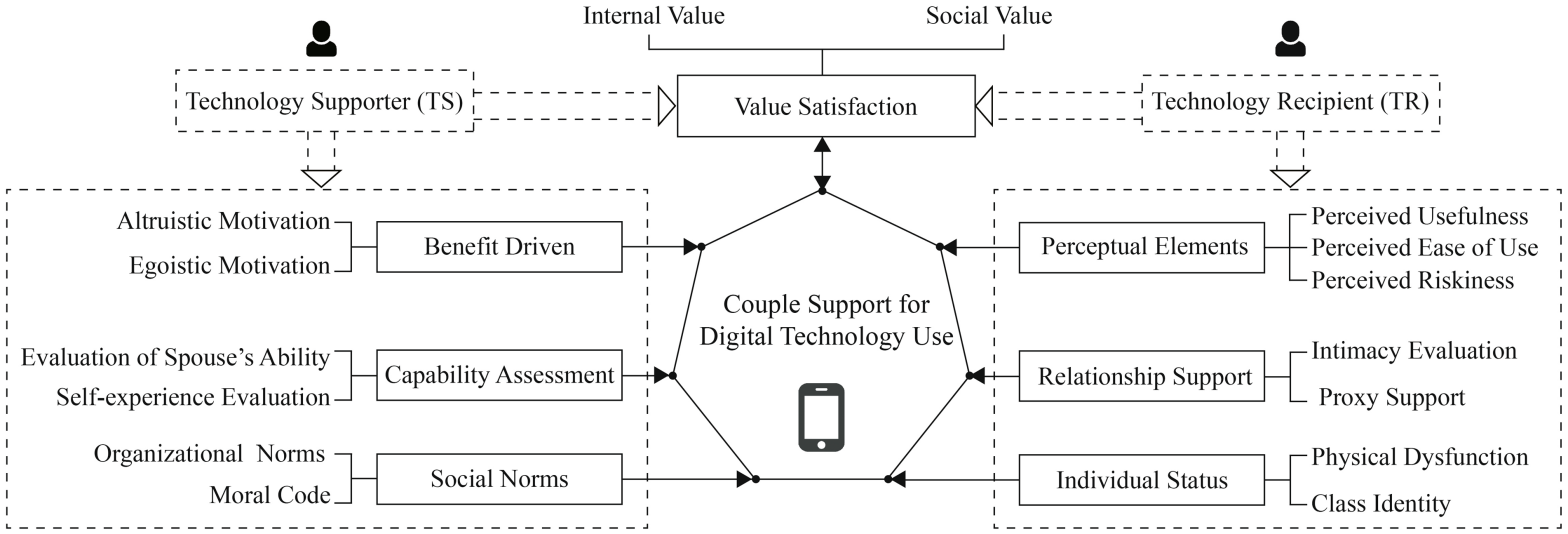
A “Motivation”model of couple support for digital technology use.

Based on the grounded theory approach and the complex dynamics among the factors discussed above, this study constructs a “Motivation” model of couple support for digital technology use (see Figure 1).

As shown in the picture, the model centers on the behavior of digital technology use support between couples and divides couples into two subjects: one is technology supporter and the other is technology recipient. The seven motivational factors that promote the occurrence of digital technology support behaviors are comprehensively analyzed from the perspective of both parties in full.

The left side of the model diagram shows that the three major motivations for technology supporters to provide technical support to their spouses are (1) social norms, (2) capability assessment, and (3) benefit driven. Similarly, the right side of the model shows that for technology recipients, the three main motivations for seeking technical support are (4) individual status, (5) relationship support, and (6) perceptual elements. The top of the model shows one motivational element that has an impact on both technology supporters and technology recipients, namely (7) value satisfaction. In particular, we show more graphically the two-way circular relationship between the affective experience of “value satisfaction” and the behavioral occurrence of “digital technology use support” through the two-way arrows. We will go deeper into the theoretical model in the discussion section.

## 5. Discussion

For older adults, multiple factors influence the consideration of whether to provide digital technology use support for spouses, or whether to seek support. The findings of psychological motivations in digital technology use support behaviors among the 15 couples in this study provide new evidence for dynamic research in this area. Here, we discuss how these psychological motivation findings have enhanced our understanding of older couples’ technology use support behaviors, as well as limitations of the current study and recommendations for future research.

### 5.1. Theoretical implications

First, we built a “Motivation” model of couple support for digital technology use among older adults. Previous literature has also attempted to interpret the occurrence of digital technology support behaviors among older adults mechanisms. For example, Hunsaker et al. (2020) summarized the reasons why older adults who are technology supporters provide digital media support to their peers around older adults; Marler and Hargittai (2022) found that the motivational factors of older adults as technology seekers when seeking digital technology support from their partners included convenience, expertise and social dynamics factors. However, few studies have examined the psychological motivational factors in the interaction between older couples by dividing them into two different types of subjects when focusing on technical support behaviors between older couples. Therefore, to fill the gap in the previous literature, our model explains the motivation to offer support to spouses in older couples’ digital technology use, as well as the motivation to seek support. Through the construction of this model, we contribute to the research of technical support behaviors among couples in the field of active aging.

Second, our study shows that older adults have the ability to act as technology supporters and provide technical support to their spouses. Digital technology support behaviors occurred between 11 of the 15 couples we interviewed. We drew on Self-determination theory (Deci and Ryan, 1985) and proposed main motivational elements. In particular, our study confirms that elements of organizational normative motivation are taken into account by older technology supporters. The finding is consistent with that of Hunsaker et al. (2020) who found that “not being required by external social norms to provide technology support” was one of the reasons why older adults were unable to provide support to their peers. This may be because under the influence of China’s collectivist culture (Earley, 1993) people will abide by and respond to the normative requirements of the organization, and when social and organizational norms are not yet in place, individuals will lack the corresponding initiative. Furthermore, we highlight the impact of altruistic motivation on older adults when providing digital technology support. This also accords with the observation of some previous scholarly studies, which showed that older adults are influenced by altruistic motivation to volunteer (CHEUNG et al., 2006; Principi et al., 2012; Chen and Morrow-Howell, 2015). This finding may be explained by the socioemotional selectivity theory (Carstensen et al., 1999), which argues that as people age, they tend to be more likely to engage in altruistic activities (Carstensen and Hershfield, 2021).

Third, we learned about the motivational factors that older adults consider when seeking technical support, especially some of the limiting factors which may, to some extent, cause the hesitation of older adults to seek support from their partners. Previous literature has also attempted to understand such constraints. For example, Hunsaker et al. (2019) found that older adults often perceive themselves as having the same technical knowledge as their spouse, which may prevent them from seeking help from their spouse. Our study adds the influence of individual state motivation and integrates evaluation factors such as education (Gonçalves et al., 2017), age (Vroman et al., 2015), income, and social capital (Petrovčič et al., 2022) in individual state motivation. Our study found that in rural China, older adults, who are digitally disadvantaged, are often limited by these factors and perceive individual status as insufficient, thus not actively seeking support from their spouses. In addition, we discuss the phenomenon of “proxy support” among the digitally disadvantaged older adults. Previous research on “proxy internet use” (PIU) and “users-by-proxy” has provided some ideas for this study. For example, Reisdorf et al. (2021) research has suggested that people with higher operational skills are also more likely to provide PIU. Our study demonstrates that older adults seek out spouses with higher relative skill levels than theirs as proxies. This echoes the findings of a recent study by Grošelj et al. (2022) that spouses appear to have become an important resource for active participation in PIU in an aging population. Meanwhile, according to the TAM (Davis, 1989), we combined three aspects of perceived usefulness, perceived ease of use, and perceived riskiness to fully reveal the impact of perceived motivation on older adults seeking technical support. This finding suggests that it is a pressing issue to enhance the motivation of older adults to seek help by improving their perception elements of technology in technical support.

Fourth, drawing on socioemotional selectivity theory (Carstensen et al., 1999), we propose that the “value satisfaction” motivation and “digital technology use support behavior” are mutually reinforcing. Both spouses can reap value satisfaction from digital technology-enabled behaviors, and this value satisfaction can also facilitate the reoccurrence of future digital technology-enabled behaviors. The study also echoes Bardach et al. (2021) call to action that when encouraging older adults to embrace and adopt technology, such technology use should be emphasized as beneficial to meeting emotional goals and needs. More importantly, we highlight the value satisfaction that older adults, as technology supporters, can also derive from providing such support in terms of respect, a sense of accomplishment, and satisfaction (Hunsaker et al., 2020).

### 5.2. Limitation and directions for future research

This study has some limitations and opens up avenues for future research. First, although our study opens a window into digital technology support for older couples in China, our study may have limitations compared to a broader population. Therefore, future studies could be conducted among older adult populations in different countries to capture a greater diversity of motivations.

Second, our study was unable to conduct a longitudinal analysis of older couples’ digital technology support behaviors. In the future, the changing dynamics of digital technology support can be better captured by observing older adults who differ in the occurrence and duration of digital technology support behaviors. For example, our current interview capture that older adults who are technology recipient, after receiving spousal technology help and acquiring digital skills, begin to take on the role of technology supporters to help their peers around them (e.g., M-7-SXW). Therefore, we suggest that future research could focus on how digital technology use support behaviors can help older adults move from being technology recipient to technology supporter in order to reduce the digital divide among older adults of the same age.

Third, future qualitative and quantitative studies can build on our findings by focusing on the different dimensions of value satisfaction that older couples reap from digital technology use support behaviors to better promote digital technology use and value realization among older adults.

## 6. Conclusion

The issue of digital technology use support among rural old couples deserves the attention of academics and policymakers to narrow the digital divide between urban and rural areas and promote healthy aging in underdeveloped areas.

### 6.1. Contributions

The main contribution of this paper are twofold: First, this study focuses on the important role of domestic spouse support on the digital technology of older adults, who are no longer just a vulnerable group within the family but can also be a provider of technology support. Second, previous studies have examined the influencing factors of digital media use and digital literacy enhancement among older adults, while the association and paths of action among the elements have not been well answered. In this study, based on summarizing and concluding the psychological factors of digital technology support behaviors among older couples, we further elucidated the relationships and constructed a theoretical model. More specifically, the identification of these influencing elements from both technology supporter and technology recipient can help in advancing rural older couples to blend into digital society by promoting more general technology support between couples.

### 6.2. Practical implications

Based on the results discussing above, we propose the following practical implications to improve older adults’ digital adoption.

First, government has the function of taking action and providing social support (Hale et al., 2021) and plays an important role in facilitating the occurrence of digital technology behaviors among older couples and effectively address the difficulties of using smart technology for the elderly. From the perspective of motivating factors for digital technology support behaviors among elderly couples, the government should do the following: (1) Play a guiding role in the government’s digital technology support for the elderly, and formulate policies to advocate digital technology support behaviors among elderly couples. (2) Strengthen the construction of infrastructure such as fifth generation mobile communication technology and fiber optic broadband in rural areas, so as to clear the infrastructural barriers to digital life for the elderly. (3) Integrate the development of digital literacy into family and social education, thereby providing opportunities for older adults to continue learning.

In addition, the community is an important place for older adults to live and should be considered in the following ways: (1) Develop digital technology support activities around older couples. For example, conduct digital skills mutual help training sessions around digital skills between older couples to enhance communication between older couples. (2) To make the elderly aware of the importance and convenience of digital technology (to improve perceived usefulness and perceived ease of use) by holding lectures and other forms. (3) Prepare and distribute manuals on the use of digital technology to enhance the ability of elderly people to identify illegal acts such as Internet rumors and telecommunication network fraud (reduce perceived risk).

More importantly, the couple is important subject in the occurrence of technical support behaviors of older adults in the home. An interactive unit is formed between older couples around digital technology support. Therefore, elderly couples can make the following efforts: (1) elderly people with higher digital technology level in the family should take the initiative to provide technical support to their spouses (2) elderly people who are the disadvantaged group of digital technology should be optimistic about their individual status and take the initiative to seek help from their spouses so as to enjoy the convenience brought by the development of digital society. (3) Both spouses should improve communication, help each other and improve together in the use of digital technology to promote the realization of their self-worth.

## Data availability statement

The original contributions presented in the study are included in the article/supplementary material, further inquiries can be directed to the corresponding author.

## Ethics statement

The studies involving human participants were reviewed and approved by the Institutional Review Board of Shanghai Jiao Tong University. The patients/participants provided their written informed consent to participate in this study.

## Author contributions

JM: study design, data collection, data analysis, and paper writing. JC: paper writing, manuscript review, and editing. QZ: data analysis and supervision. All authors contributed to the article and approved the submitted version.

## Conflict of interest

The authors declare that the research was conducted in the absence of any commercial or financial relationships that could be construed as a potential conflict of interest.

## Publisher’s note

All claims expressed in this article are solely those of the authors and do not necessarily represent those of their affiliated organizations, or those of the publisher, the editors and the reviewers. Any product that may be evaluated in this article, or claim that may be made by its manufacturer, is not guaranteed or endorsed by the publisher.

## Footnotes

^1^See http://www.pewinternet.org/fact-sheet/internet-broadband/

^2^See https://china.unfpa.org/sites/default/files/pub-pdf/The%20Youth%20Population%20in%20China%20%282018%29-CN_1.pdf

^3^See http://agri.ckcest.cn/resourcenavigation/webinfo/e5115d60-1b3a-4af7-801a-dbb782421aef.html

^4^See http://www.wuzhi.gov.cn/wzx/cms/20220426031853000001

^5^Grass-roots mass self-governance organizations are villagers’ committees established in China in rural areas according to the areas where the residents live, which are self-management, self-education and self-service organizations for rural villagers.
